# Polymorphism rs7278468 is associated with Age-related cataract through decreasing transcriptional activity of the *CRYAA* promoter

**DOI:** 10.1038/srep23206

**Published:** 2016-03-17

**Authors:** Xiaoyin Ma, Xiaodong Jiao, Zhiwei Ma, J. Fielding Hejtmancik

**Affiliations:** 1 Laboratory of Developmental Cell Biology and Disease, School of Ophthalmology and Optometry and Eye Hospital, Wenzhou Medical University, 325003, China; 2 Ophthalmic Genetics and Visual Function Branch, National Eye Institute, National Institutes of Health, Bethesda, MD 20892, USA

## Abstract

CRYAA plays critical functional roles in lens transparency and opacity, and polymorphisms near CRYAA have been associated with age-related cataract (ARC). This study examines polymorphisms in the *CRYAA* promoter region for association with ARC and elucidates the mechanisms of this association. Three SNPs nominally associated with ARC were identified in the promoter region of *CRYAA*: rs3761382 (P = 0.06, OR (Odds ratio) = 1.5), rs13053109 (P = 0.04, OR = 1.6), rs7278468 (P = 0.007, OR = 0.6). The C-G-T haplotype increased the risk for ARC overall (P = 0.005, OR = 1.8), and both alleles and haplotypes show a stronger association with cortical cataract (rs3761382, P = 0.002, OR = 2.1; rs13053109, P = 0.002, OR = 2.1; rs7278468, P = 0.0007, OR = 0.5; C-G-T haplotype, P = 0.0003, OR = 2.2). The C-G-T risk haplotype decreased transcriptional activity through rs7278468, which lies in a consensus binding site for the transcription repressor KLF10. KLF10 binding inhibited CRYAA transcription, and both binding and inhibition were greater with the T rs7278468 allele. Knockdown of *KLF10* in HLE cells partially rescued the transcriptional activity of *CRYAA* with rs7278468 T allele, but did not affect activity with the G allele. Thus, our data suggest that the T allele of rs7278468 in the *CRYAA* promoter is associated with ARC through increasing binding of KLF-10 and thus decreasing *CRYAA* transcription.

Age-related cataract (ARC), is defined as a lens opacity that occurs after age 45 or 50^1^. The prevalence of ARC increases with age, and it is the major cause of blindness in advanced countries. The number of patients with ARC in American is estimated to increase to more than 30 million by 2020^2^,^3^. ARC is a multifactorial disease, and both genetic and environmental factors influence risk for it. Most genetic risk factors demonstrated to date have been the single nucleotide polymorphisms (SNPs), although these have not been shown to be causative themselves. A number of SNPs and genes have been reported to be associated with ARC (see Cat-Map)^4^, including those in CRYAA^5^ and EPHA2^6^,^7^, and a meta-analysis identified two SNPs near the CRYAA and KCNAB1 genes that were significantly associated with ARC^8^.

In addition to being a major structural protein component of the lens, CRYAA (αA-crystallin) can protect other crystallins against thermally induced inactivation or aggregation and is thus critical for the maintenance of lens transparency over time^9^,^10^. CRYAA can protect lens cells against heat and oxidative stress-induced cell death^11^, and it has been suggested that, through trapping aggregation-prone denatured protein, CRYAA can delay the development of ARC^12^. Mutations in *CRYAA* have been shown to cause congenital cataracts (see *Cat-Map*)^4^, and the SNP rs766604319 in the *CRYAA* gene has also been reported to be associated with ARC^5^. *Cryaa* knockout mice display a small lens, lens epithelial cell death, reduced proliferation, cataract, and inhibition of pathological neovascularization^12^,^13^,^14^. The expression level of *CRYAA* was reported to be decreased in ARC patients’ lenses^8^,^15^, although the mechanisms of the *CRYAA* transcriptional regulation in ARC lens have not been fully elucidated. In addition, a previous paper has suggested association of a synonymous G > A transition c.6G > A with ARC^5^. Taken together, these reports suggest that CRYAA might be an excellent candidate for contributing to ARC.

The DNA sequence of the promoter region is a major determinant of gene expression. Functional noncoding SNPs in a promoter or enhancer region have been shown to influence transcriptional activity^16^,^17^,^18^, but the relationship of such functional noncoding SNPs with ARC is currently unknown. In order to explore this question, 1kb of the *CRYAA* promoter region was sequenced in Northern Italian cataract patients and compared with ethnically and age-matched unaffected control individuals. We identified three SNPs that show association with ARC in this population. These SNPs are in strong linkage disequilibrium, and the haplotypes of these SNPs are also associated with ARC. To analyze the possible functional roles of these SNPs in *CRYAA* transcription further, we examined the effects of the individual SNPs, and the results show that rs7278468 has the greatest effect on the transcriptional activity of *CRYAA*. This SNP is located in the binding motif of the transcriptional repressor KLF10 (Kruppel-like factor 10, also named TIEG, or TGFβ-inducible early gene), and a single-base change from the G to T allele of rs7278468 enhanced the binding affinity of KLF10 and thus inhibited the transcription of *CRYAA*. Knockdown of *KLF10* in HLE cells can partially rescue the transcriptional activity of *CRYAA* with rs7278468 T allele but has no effect on the G allele. Our results thus suggested that the rs7278468 T allele in the *CRYAA* promoter decreases its transcriptional activity through enhanced binding of KLF10 which would be expected to increase susceptibility to ARC.

## Results

### Identification of polymorphisms in *CRYAA* promoter region and association with age-related cataract

In order to identify polymorphisms in *CRYAA* promoter region that might be associated with age-related cataract (ARC), the *CRYAA* promoter regions (−1 to −1000) of 215 ARC patients and 106 normal control individuals were sequenced. Three polymorphisms were identified in the *CRYAA* promoter region of both patients and control individuals: rs3761382, rs13053109 and rs7278468 (Supplementary Fig. S1). All three of these polymorphisms show a statistically significant or suggestive association with all ARC: rs3761382 (P = 0.03, OR = 1.5, 95% CI = 1.1–2.1), rs13053109 (P = 0.06, OR = 1.5, 95% CI = 1.1–2.0), rs7278468 (P = 0.002, OR = 0.6, 95% CI = 0.5–0.8) (Table 1). When specific subtypes of cataract were tested, these SNPs have a stronger association with cortical cataract: rs3761382 (P = 0.0008, OR = 2.1, 95% CI = 1.4–3.1), rs13053109 (P = 0.002, OR = 2, 95% CI = 1.3–2.9), rs7278468 (P = 0.0002, OR = 0.5, 95% CI = 0.4–0.7) (Table 2). All three SNPs are in HWE in the control group (P > 0.05).

**Figure 1:**
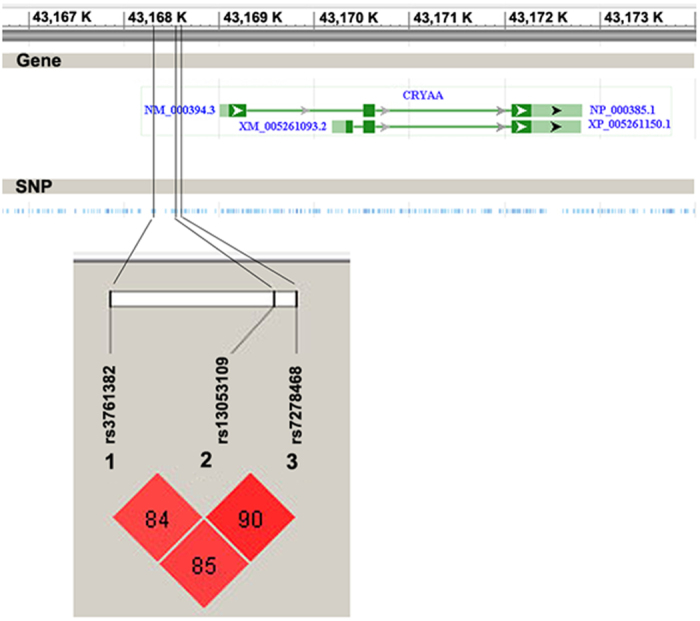
LD status of the 3 SNPs in *CRYAA* promoter region. Each diamond represents a pair-wise comparison between the 2 SNPs, and the respective D′ is given within each square. Higher levels of red shading indicate higher values of D′, with the maximum being 100%.

Linkage disequilibrium among these SNPs was analyzed by Haploview, and showed strong linkage disequilibrium (all D′ values > 0.8, Fig. 1), so that the haplotypes formed by the three SNPs were tested for association with ARC. The C-G-T haplotype showed strong association with ARC overall (corrected P = 0.001, OR = 1.8, 95% CI = 1.3–2.4), while the T-C-G haplotype showed a suggestion of being protective (corrected P = 0.05, OR = 0.7, 95% CI = 0.5–0.9, Table 3). The odds ratios obtained with these haplotypes were similar to those seen with the individuals SNPs. As seen with the individual SNPs, the C-G-T haplotype was more strongly associated with cortical ARC (corrected P = 0.00008, OR = 2.2, 95% CI = 1.5–3.3), and the T-C-G haplotype also was more protective for cortical ARC (corrected P = 0.001, OR = 0.5, 95% CI = 0.3–0.7, Table 4).

### The C-G-T haplotype reduces *CRYAA* transcriptional activity

**Figure 2:**
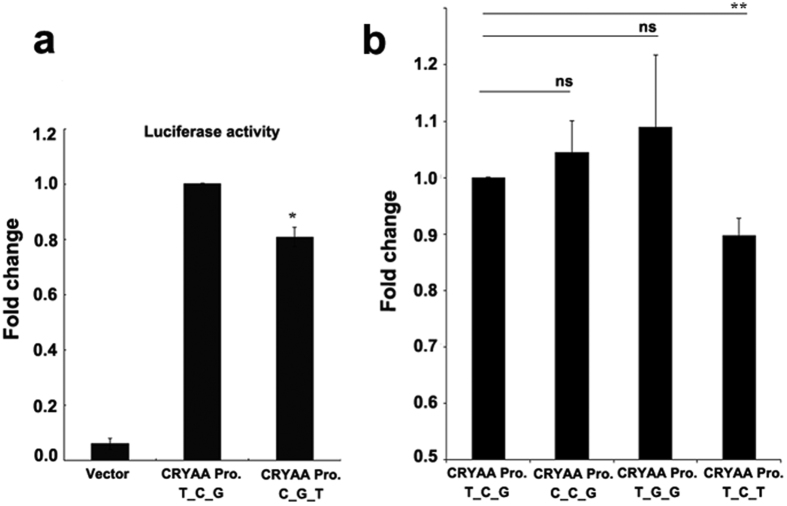
ARC associated allelic variation in rs7278468 alters the transcriptional activity of *CRYAA*. (**a**) The *CRYAA* promoter containing either the T-C-G or C-G-T haplotype was transfected into HLE cells and Luciferase activity was measured 72 hours after transfection. (**b**) Site–directed mutagenesis was carried out to separate the effects of rs3761382, rs13053109 and rs7278468. Promoter activity was tested in HLE cells 72 hours after transfection. The rs7278468 T allele reduced *CRYAA* promoter activity compared with the rs7278468 G allele. Results are shown as mean ± SD. * Indicates P < 0.05, ** indicates P < 0.01. Experiments were repeated 3× in Fig. 2A and 4× in Fig. 2B.

The location of these 3 SNPs in the promoter region of *CRYAA* suggested that they might affect transcriptional regulation of the gene. In order to address this question, we constructed a luciferase reporter vector containing the *CRYAA* promoter with either the risk haplotype (C-G-T) or protective haplotype (T-C-G). Transcriptional activity of the *CRYAA* promoter was estimated using a dual-luciferase reporter assay 72 hours after transfection of HLE cells. The *CRYAA* promoter has transcriptional activity in HLE cells: compared with the vector, the luciferase activity was more than 10 times higher in cells transfected with the vector containing the *CRYAA* promoter (Fig. 2). When transcriptional activity of the *CRYAA* promoter with the protective T-C-G haplotype was normalized to 1.0, the transcriptional activity of the promoter with the C-G-T risk haplotype was decreased to about 80% (Fig. 2a). This data demonstrated the risk haplotype of C-G-T reduced *CRYAA* transcriptional activity in HLE cells and suggested this haplotype might induce ARC through decreasing the transcriptional level of *CRYAA*.

### rs7278468 Is largely responsible for altering transcriptional activity of the *CRYAA* promoter

While the previous data implicated the SNP haplotypes in *CRYAA* promoter influence transcriptional activity, they did not clarify which individual SNP(s) was most responsible for regulating the transcriptional activity of *CRYAA*. In order to address this question, we carried out site–directed mutagenesis to alter each individual SNP from the allele present in the protective haplotype to that present in the risk haplotype and analyzed the resulting transcriptional activity of the *CRYAA* promoter. As can be seen in Fig. 2b, 72 hours after transfection with the luciferase reporter plasmid, there was no significant difference between transcriptional activities of the promoters containing the individual risk alleles of rs3761382 and rs13053109 occurring as a single base variation when compared with the protective haplotype T-C-G. However, when the single base of rs7278468 was changed from the G to the T allele, transcriptional activity of the resulting *CRYAA* promoter with the T-C-T haplotype decreased about 12%, significant with a P < 0.01. This suggests that the sequence change seen in rs7278468 might directly affect CRYAA expression, while rs13053109 and rs3761382 show association with ARC through linkage disequilibrium with rs3761382.

### KLF10 inhibits *CRYAA* transcription through rs7278468 T allele

While the above results showed that rs7278468 alleles directly affect *CRYAA* transcriptional activity, the mechanism through which this might occur remained unclear. Since rs7278468 lies 361 bp upstream of the transcription start site and does not reside within the actual consensus promoter itself, it seemed possible that it might affect binding of one or more transcription factor(s) and affect the transcriptional activity of *CRYAA* in that fashion. We analyzed transcription factor binding sites in the *CRYAA* promoter region using the Genomatix system (https://www.genomatix.de). Based on these results, rs7278468 lies in the binding motifs of several transcription factors including KLF10 (Kruppel-like factor 10) (Supplementary Table S1; Supplementary Fig. S2a). KLF10 is a ubiquitously transcription repressor that is known to be an effector in the TGF beta (transforming growth factor, beta) signaling pathway^19^.

**Figure 3:**
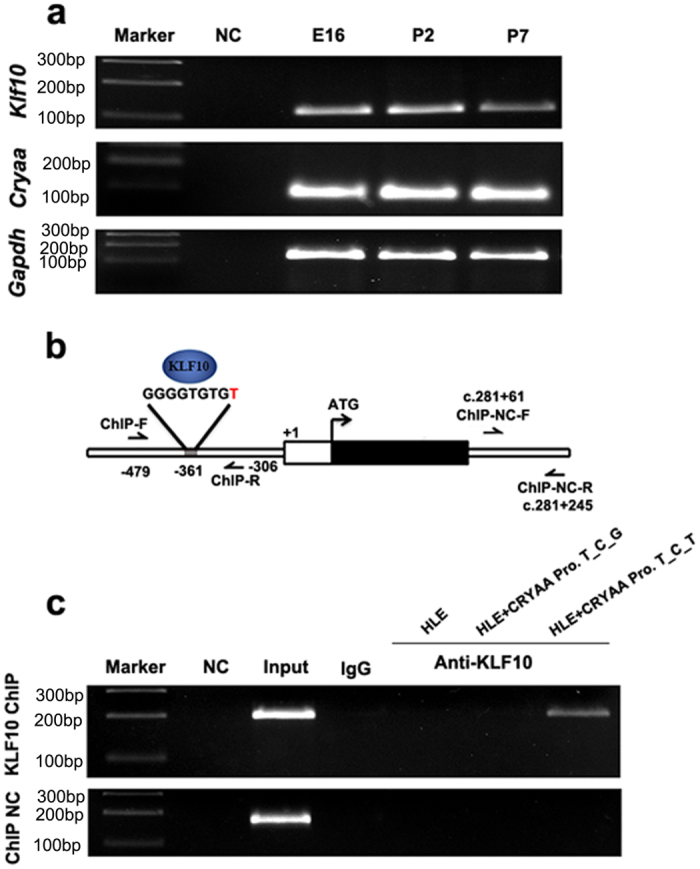
The ARC associated rs7278468 T allele increased the binding affinity of KLF10 to the *CRYAA* promoter. (**a**) RT-PCR demonstrated expression of *Klf10* and *Cryaa* mRNA in the mouse lens at various ages. *Gapdh* was used as endogenous control. (**b**) Diagram of the *CRYAA* gene promoter region showing the KLF10 binding motif containing rs7278468 (shown in red). ChIP-F and ChIP-R show the region for ChIP PCR and ChIP-NC-F and ChIP-NC-R are primers used for the negative control. (**c**) ChIP PCR was carried out using HLE cells or HLE cells 48 hours after transfection with the *CRYAA* promoter having different rs7278468 haplotypes. Input is total genomic DNA as positive control, and IgG is the negative control for nonspecific binding. A PCR band was only detected in HLE cell transfected with T_C_T CRYAA promoter. Gels showing RT-PCR (a) were all 2% TBE agarose gels and those showing ChIP (c) were 4% TBE agarose gels. Gel photographs are cropped and the originals are available in the supplementary data.

The TGF-beta signaling pathway plays functional roles in inducing cataract and has been reported to inhibit *CRYAA* transcription, even though the precise mechanisms have not been fully elucidated^20^,^21^. This, along with its expression and the complete match of the consensus sequence, made KLF10 a good candidate for mediating the effects of the rs7278468 on CRYAA expression. In order to clarify whether KLF10 regulates the transcription of *CRYAA* in the lens, we initially examined *Klf10* expression in the C57BL/6 mouse lens. Total RNA was isolated from E16.5, P2 and P7 mice, and *Klf10* mRNA expression was examined by RT-PCR. *Klf10* was seen to be expressed in mouse lens at all time points, perhaps somewhat higher in the embryonic period (Fig. 3a). *KLF10* is also expressed in the HLE cells (Supplementary Fig. S2b), which have a GG genotype for rs7278468 (Supplementary Fig. S3). In order to identify whether KLF10 can regulate *CRYAA* through binding to rs7278468, ChIP-PCR was used to analyze KLF10 binding affinity in HLE cells (Fig. 3b). With the ChIP-NC primers a strong band was detected in the Input lane, but not in any immunoprecipitated sample. Similarly, with the ChIP-PCR primers, no ChIP-PCR band was detected in HLE cells alone or in HLE cells transfected with the *CRYAA*_T-C-G plasmid. However, a ChIP-PCR band was detected in the Input and *CRYAA*_T-C-T transfected cells (Fig. 3c). This result demonstrated that KLF10 binds directly to the CRYAA promoter, that the rs7278468 T allele can enhance this binding, and suggested that KLF10 might inhibit *CRYAA*’s transcription through direct interaction with the promoter, including sequences overlapping the rs7278468 T allele.

### Knockdown of KLF10 in HLE cells partially rescues transcription of *CRYAA* with rs7278468 T allele

**Figure 4:**
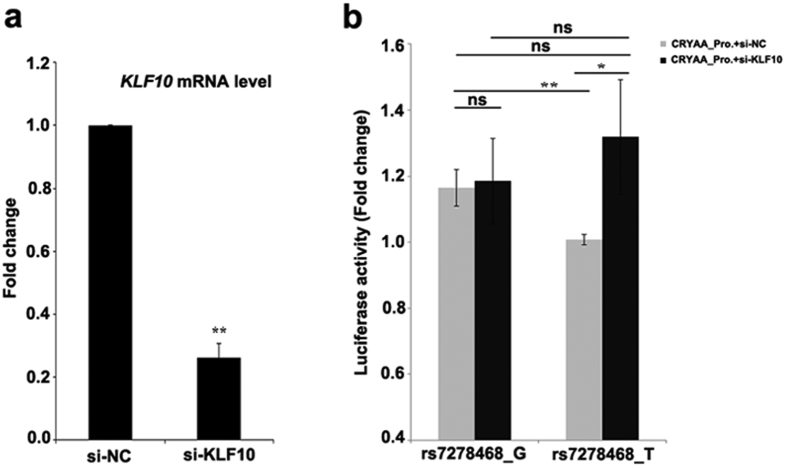
Knockdown *KLF10* in HLE cells rescued the *CRYAA* transcriptional activity of the rs7278468 T allele. A promoter: specific siRNA was used to knock down *KLF10* in HLE cells and knock down efficiency was estimated by real-time PCR 48 hours after transfection. B: Luciferase activity assay in *KLF10* knock down cells. The *CRYAA* promoter with the rs7278468_T or rs7278468_G was transfected into HLE cells along with si-NC or si-KLF10, and the luciferase activity was measured 48 hours after transfection (the luciferase activity of rs7278468_T was normalized as 1.0). Data is shown as mean ± SD. * indicated P < 0.05 and ** indicated P < 0.01.

In order to confirm that the rs7278468 T allele decreases *CRYAA* transcription through increasing binding of KLF10 and thus decreasing transcription of CRYAA, we tested whether knockdown of *KLF10* in HLE cells could rescue the transcription of the T rs7278468 allele *CRYAA* promoter. Transfection of HLE with si-KLF10 knocks down expression of *KLF10* by about 75% (Fig. 4a, Supplementary Fig. S4). When compared to samples co-transfected with the si-NC control, knock down of *KLF10* in HLE cells had little or no effect on transcription of the *CRYAA* promoter containing the rs7278468 G allele. In contrast, while the transcriptional activity of the *CRYAA* promoter with the rs7278468 T allele was 16.5% below that of the rs7278468 G allele promoter, when co-transfected with the si-NC control plasmid (p < 0.01), knocking down *KLF10* increased in luciferase activity of the *CRYAA* promoter with the rs7278468 T allele by about 35% (p < 0.05, Fig. 4b). The *CRYAA* transcription activity with rs7278468 T allele in si-KLF10 knock down cells was not significantly different from that with the rs7278468 G allele transfected with either the si-NC plasmid or with si-KLF10. This confirms that binding of KLF10 to the *CRYAA* promoter region inhibits its transcriptional activity, and the rs7278468 T allele decreases *CRYAA* transcription at least in part by increasing binding of KLF10 to the *CRYAA* promoter.

## Discussion

**Figure 5:**
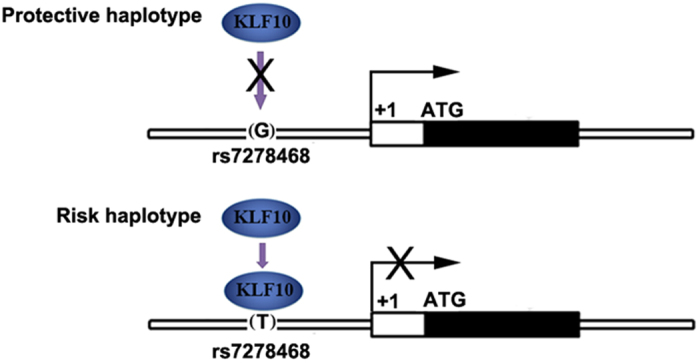
Model of the mechanism of KLF10 regulation of *CRYAA* transcription and the effect of rs7278468. in the presence of the rs7278468 G allele, KLF10 does not bind to the promoter region of *CRYAA*, and it will not inhibit the transcription of *CRYAA*. In the presence of the rs7278468 T allele KLF10 binds to the recognition motif in the promoter and inhibits the transcription of *CRYAA*.

Taken together, our results suggest that the rs7278468 T allele is associated with ARC, and that this single base change is causal, in that the *CRYAA* promoter region is sufficient to alter the activity of its transcription significantly. rs7278468 Lies in the binding motif of KLF10. In the presence of the rs7278468 T allele, KLF10 can bind to the *CRYAA* promoter efficiently and inhibit its transcription, while in the presence of the rs7278468 G allele, binding is decreased and KLF10 does not appear to affect the transcription of *CRYAA* significantly (Fig. 5). When *KLF10* is knocked down in HLE cells, the transcriptional activity of the *CRYAA* promoter with the rs7278468 G allele is unaffected, but the activity of the *CRYAA* promoter with T allele is increased significantly to levels seen with the G allele, consistent with the genetic and molecular data implicating the rs7278468 T allele in increasing risk of ARC through reducing CRYAA transcriptional activity and CRYAA expression.

CRYAA is highly expressed in the lens and plays important roles in both the development and maintenance of lens clarity. *CRYAA* knockout mice develop small lenses with opacities initially confined to the nucleus but progressing to total cataract^13^. Light and electron microscopy of *CRYAA* knockout lenses show dense inclusion bodies. Mutations that severely disrupt the α-crystallin protein structure are well known to cause congenital cataracts, while mutations with milder effects have been implicated in later onset and progressive cataracts^22^,^23^,^24^,^25^,^26^. In addition, levels of αA-crystallin have been reported to be decreased in age related nuclear cataract lenses^27^, and CRYAA mRNA was shown to be decreased in lens epithelia from ARC patients^27^, although the latter was correlated with hypermethylation of the CRYAA promoter. However, the decrease in αA-crystallin protein was not confirmed by a second study^28^. In addition, the well described activity of α-crystallin as a lens chaperone provides a logical basis for its role in protecting the lens from ARC^10^,^29^. These findings, along with the previous report suggesting association of a synonymous G > A transition c.6G > A with ARC emphasize the importance of functional α-crystallin for lens transparency and make CRYAA an excellent candidate for contributing to ARC.

A gene’s promoter, the key element for its transcriptional regulation, consists of a core promoter sequence as well as enhancer and silencer sites. Trans-factors including transcription factors can bind to consensus motifs and modify the activity of the core promoter. Variation in binding motifs can affect binding affinities of the corresponding factors, and there are several studies showing that SNPs in promoter or enhancer region can induce a novel phenotype by affecting a gene’s transcriptional activity^16^,^17^,^18^. However, to date no functional noncoding SNP in a promoter region has been implicated in inducing cataract through regulating transcriptional activity. As an important lens protein, *CRYAA* has been demonstrated to be regulated by transcription factors including PAX6, c-Maf and CREB^30^, but the complete transcriptional regulation of *CRYAA* has not been delineated. In this work we demonstrated that *CRYAA* is negatively regulated by KLF10 through binding to its consensus motif in the CRYAA promoter. The T allele of the rs7278468 polymorphism in KLF10’s binding motif increases its binding affinity, decreasing CRYAA transcription. It seems likely that the resulting decrease in CRYAA transcription yields lower levels of αA-crystallin protein in the lens, and thus contributes to the increased frequency of age related cataract in individuals having the rs7278468 T allele.

KLF10 has been reported to function in inhibiting proliferation and inducing apoptosis. *Klf10* knockout mice display Osteopenia and Hypertrophic cardiomyopathy^31^, but a functional role for KLF10 in the lens has not been reported previously. However, it is known that KLF10 is a zinc finger transcription factor that mediates TGF-beta signaling^19^. TGF-beta previously has been implicated in cataractogenesis: TGF-beta intravitreal injection can induce rat cataract^32^, and overexpression of TGF-beta in the mouse lens induces cataract^21^. TGF-beta has been demonstrated to inhibit FGF induced transcriptional activity of CRYAA both *in vitro* and *vivo*^20^, although the precise mechanism was not elucidated. Our current data show that KLF10 is expressed in the mouse lens and can inhibit transcription of *CRYAA*, consistent with the previous results, suggesting a mechanism for TGF-beta inhibition of *CRYAA* transcription. The functional roles of KLF10 in the lens and cataractogenesis are currently under further investigation.

Identification of the genetic risk factors for ARC is becoming increasingly important for a number of reasons. First, identified genes and their related pathways are potential therapeutic targets for prevention or delay of ARC. Based on the important functional roles of CRYAA and TGF beta signaling pathway in the lens and especially in cataract, rs7278468 or TGF-KLF10 signaling pathway might provide therapeutic targets to delay the development of cataract. In addition, simply identifying individuals at risk for ARC can increase the efficiency of screening the population for incipient opacities in the lens in order to identify potential candidates for therapeutic intervention. It is even possible that a sufficient increase in risk identified on the basis of genetic testing alone might justify preventative intervention in some individuals, if the intervention was benign enough. The combination of an anticipated doubling of the need for cataract surgery in the next 20 years^33^, and the estimate that delaying the development of cataract by 10 years would decrease the need for cataract surgery by about 45%^34^, with recent advances suggesting the possibility of preventative therapy for ARC^35^ mean that identifying the genetic architecture and mechanisms underlying susceptibility to ARC could have an impact on clinical patient care in the near future.

In this study, we identified three SNPs in the CRYAA gene promoter region that are associated with ARC both in allele or the haplotype analysis. The C-G-T haplotype appeared to be a risk factor for ARC while T-C-G seemed to be protective. Analysis of the transcriptional activity of the CRYAA promoter with different SNP alleles in HLE cells demonstrated that the rs7278468 T allele to be responsible for the reduced transcriptional activity imparted by the original risk haplotype. rs7278468 Lies in the recognition motif for KLF10, and the T allele increases KLF10 binding and thus inhibits CRYAA transcription. Combined with previously published data regarding CRYAA levels and ARC, 8 this suggests that rs7278468 induces ARC through decreasing transcription of CRYAA’s and thence the level of αA-crystallin protein in the lens of ARC patients. Over time, this decrease in αA-crystallin might combine with environmental damage and aging to decrease the buffering capacity in the lens for denatured proteins and lead to ARC.

## Methods

### Subjects

This study included a total of 382 individuals, 215 with age-related cataract and 167 age-matched normal control individuals. All the individuals over 50 years of age from Northern Italy were ascertained from the Clinical Trial of Nutritional Supplements and Age-Related Cataract Study. Detail information of the patient population has been described in our previous papers^6^,^36^,^37^. Briefly, individuals with an age-related cataract in at least one eye were defined as having a cataract, while individuals with no cataract in either eye were considered to be controls. Patients with limited life expectancy and patients with cataract secondary to identifiable causes, such as diabetes, trauma, and steroid administration, were excluded. Cataract status was determined by slit lamp examination using the Lens Opacities Classification System II (LOCSII). This system provides ordinal scores for grading the presence and severity of nuclear opalescence (scale, 0 to 4), cortical opacities (scale, 0 to 5), and posterior subcapsular opacities (scale, 0 to 4). For this study, a specific opacity was judged to be present when the grade was 1 or greater in at least one eye. A person’s grade for each opacity type was the more severe grade of the two eyes. A person was. classified as having no lens opacities if he or she had a grade of 0 for each type of opacity in both eyes. The age range of individuals with ARC is 50–86, with an average age of 75.7, while the age range of the normal control individuals is from 61–86, with an average age of 74.5. Forty nine percent of the control individuals were female, as were 59% of all cataract patients and 67% of the cortical cataract patients. Ethical approval for this study was obtained from the University of Parma and the NIH CNS IRBs, all methods were carried out in accordance with the approved guidelines, and written informed consent was given in accordance with the tenets of the Declaration of Helsinki.

### DNA samples

Genomic DNA was isolated from human blood samples using a standardized protocol that included cell lysis with anionic detergent, high salt precipitation of proteins, ethanol precipitation to concentrate DNA followed by further purification of DNA with a buffered phenol/chloroform mixture^38^. After a final ethanol precipitation the DNA pellet was dissolved in 10 mM Tris-1 mM EDTA, pH 8.0

### PCR and sequencing

1kb of *CRYAA* promoter region was amplified and sequenced using the primers: CRYAA-Promoter-F: GGTGACACAGCAAGACTCCA; CRYAA-Promoter-R: CACGTCCATGTTCAGCTTTG. The PCR included: 10× PCR buffer: 1.0 ul, Mg^2+^ 0.6 ul, dNTP 0.5 ul, 10 pm primer 0.5 + 0.5 ul. Taq 1u, DNA 40 ng, H_2_O up to 10 ul. A touchdown PCR was performed for the first 15 cycles: 94 °C for 4 min, followed by decreasing the annealing temperature in a stepwise fashion from an initial 64 °C by 0.5 °C every second cycle, and 72 °C for 1.5 min. For the later 20 cycles: 94 °C for 40 sec, 57 °C for 30sec and 72 °C for 1.5 min and finally a prolonged elongation step at 72 °C for 10 min. PCR production was purified and analyzed by Sanger sequencing.

### Cell culture and transfection

All experiments in lens cells utilized the HLE cell line FHL124, which has 95% similarity in transcriptional profile to human lens epithelia^39^. It was kindly provided by Dr. JR Reddan (Oakland University) and cultured in 1g/L Glucose DMEM contains 10% FBS. siRNA Transfections were carried out using the PepMute reagent (SignaGen) as follows: Cells were sub-cultured 24 hours before transfection, so that the cell density reached about 80%. 1 hour before transfection, cells were cultured in fresh complete medium. In a 6-well plate, 50 nM siRNA specific to *KLF10* (si-KLF10) was mixed with 4 ul PepMute reagent into 100 ul transfection buffer. After mixing, the mixture was maintained at room temperature for about 15 min, then the transfection mixture was added to the cells, which were then cultured for 5 hours, after which the culture medium was changed with fresh complete culture medium. Forty eight hours later, the knock down efficiency or further tests were carried out. The sequence of the KLF-10 siRNA (5′ to 3′) was UUAUCCUUGAUGAAUCAAUCUGAGG (Invitrogen).

Plasmid transfections were carried out using a PolyJet Transfection Kit (SignaGen) according to the manufacturer’s protocol, in 6-well plates, 1 ug plasmid was used for transfection and 72 hours later the cells were collected for luciferase activity testing, or 48 hours after transfection the cells were used for ChIP analysis.

### Luciferase Vector construction

A 1111 bp region of the human *CRYAA* proximal promoter was amplified using CRYAA-Promoter-F and CRYAA-Promoter-R as described above. The PCR product was cloned into the PCR 2.1-TOPO (Invitrogen) vector for sequencing. After sequencing, the *CRYAA* promoter region was cloned into the PGL4.17 vector (Promega) using the restriction enzymes Hind III and EcoR V (NEB). The *CRYAA* promoter containing each SNP mutation was constructed using site–directed mutagenesis with the primersf: rs3761382-mut F: TACATCGAGGGGACGATGGCCAT, R: ATGGCCATCGTCCCCTCGATGTA; rs13053109-mut F: GGTGAGACTCTGAGGACGATGTGT, R: ACACATCGTCCTCAGAGTCTCACC; rs7278468-mut F: GGGTGTGTGCTCTCCCTCCTCT, R: AGAGGAGGGAGAGCACACACCC and confirmed by sequencing once more.

### Luciferase Reporter Assay

HLE cells were cultured in 6-well plates to approximately 80% confluency, 1ug of either PGL4-CRYAA Promoter or PGL4 plasmid and 30ng pGL4.75 [hRluc/CMV] were transfected into cells using a LipoJet Transfection Kit (SignaGen). Seventy two hours after transfection luciferase activities were checked using a dual-luciferase reporter assay system (Promega) according to the supplier’s protocol.

### RNA isolation and real-time PCR

Mouse lens or HLE cell total RNA was isolated using Trizol reagent (Life Technologies) and was reverse transcribed into cDNA using a reverse transcriptase kit (Invitrogen) with random primers. The cDNA was processed for real-time PCR using SYBR Green (Life technologies). All reactions were run in triplicate and data were normalized to *GAPDH*. The fold change relative to the control was calculated using the ΔΔCt method. All procedures with mice in this study were performed in compliance with the tenets of the National Institutes of Health Guidelines on the Care and Use Animals in Research and the ARVO Statement for the Use of Animals in Ophthalmic and Vision Research. Primers used for real-time PCR were: Human *GAPDH* F: AGGGCTGCTTTTAACTCTGGT, R: GACAAGCTTCCCGTTCTCAG. Human *KLF10* F: CCACACGGGTGAGAAGAAAT, R: CCCGCTGAGACCAAAGTTAG. And mouse *Klf10* RT-PCR primer F: GAAGTTTGCCTGTCCCATGT, R: ACTTCCATTTGCCAGTTTGG. Mouse *Cryaa* RT-PCR primer F: GAGATTCACGGCAAACACAA, R: ACATTGGAAGGCAGACGGTA. Mouse *Gapdh* F: CGTCCCGTAGACAAAATGGT, R: TCAATGAAGGGGTCGTTGAT. All PCR reactions used for RT-PCR had an efficiency greater than 95%, and single products were confirmed by melting profiles.

### Chromatin immunoprecipitation

ChIP analysis was carried in HLE cells transfected with a plasmid containing the *CRYAA* promoter 48 hours after transfection using a ChIP-IT express Enzymatic Magnetic Chromatin Immunoprecipitation kit as per the supplier’s suggested protocol (Active Motif). Antibodies used for ChIP included: Anti-Human IgG (Abcam), and Anti-KLF10 (Proteintech). Primers used for ChIP PCR were: CRYAA ChIP F: TGGTGAGTGTAACGGAGGTTC, R: CCAGGGACCATGCTAGTTCT. CRYAA ChIP NC: F2: GAGAGCGATGGACTCTGGTC, R2: GCTGATGGAGGAAAGCAAAG.

### Statistical analysis

SNP genotype frequencies, Chi square p values, odds ratios with 95% confidence intervals, and HWE (Hardy–Weinberg equilibrium) were analyzed using the SVS software package (Golden Helix, Bozeman, MT). Since the SNP haplotype extended over only 334 bp recombination was assumed to be 0 for these markers. The Chi-square P value was corrected using the Bonferroni Correction and a corrected P < 0.05 was considered to be statistically significant. The experiments of mRNA, protein and luciferase activity test were repeated three time and results presented as mean ± standard deviation (SD). The statistical significance between experimental and control groups was assessed with a Student’s t-test and a P < 0.05 was considered significant.

## Additional Information

**How to cite this article**: Ma, X. *et al*. Polymorphism rs7278468 is associated with Age-related cataract through decreasing transcriptional activity of the *CRYAA* promoter. *Sci. Rep*. **6**, 23206; doi: 10.1038/srep23206 (2016).

## Supplementary Material

Supplementary Information

## Figures and Tables

**Table 1:** Allele analysis of ARC.

SNP	Allele	Normal (%)	ARC (%)	P value	Bonf.P	OR	95% CI
rs3761382	C	67%	75.6%	0.01	0.03	1.5	1.1–2.1
	T	33%	24.4%				
rs13053109	G	69%	76%	0.02	0.06	1.5	1.1–2.0
	C	31%	24%				
rs7278468	G	67%	55%	0.0008	0.002	0.6	0.5–0.8
	T	33%	45%				

**Table 2:** Allele analysis of cortical ARC.

SNP	Allele	Normal (%)	ARC (%)	P value	Bonf.P	OR	95% CI
rs3761382	C	67%	81%	0.0002	0.0008	2.1	1.4–3.1
	T	33%	19%				
rs13053109	G	69%	81%	0.0008	0.002	2	1.3–2.9
	C	31%	19%				
rs7278468	G	67%	50.5%	0.00007	0.0002	0.5	0.4–0.7
	T	33%	49.5%				

**Table 3:** Haplotype analysis of ARC.

Haplotype	Control (%)	ARC (%)	P value	Bonf.P	OR	95% CI
C-G-T	29%	43%	0.0002	0.001	1.8	1.3–2.4
T-C-G	28%	21%	0.009	0.05	0.6	0.5–0.9
C-G-G	36%	30%	0.06	0.37	0.7	0.6–1
C-C-G	1%	2%	0.1	0.65	2.8	0.7–10
T-G-G	2%	2%	0.1	1	1.1	0.3–3.1
T-G-T	2%	1%	0.7	1	0.8	0.2–2.7

**Table 4:** Haplotype analysis of cortical ARC.

Haplotype	Control (%)	ARC (%)	P value	Bonf.P	OR	95% CI
C-G-T	30%	48%	0.00001	0.00008	2.2	1.5–3
T-C-G	28%	16%	0.0002	0.001	0.5	0.3–0.7
C-G-G	36%	30%	0.13	0.96	0.7	0.5–1
C-C-G	1%	3%	0.07	0.55	3.2	0.8–13
T-G-G	2%	2%	0.75	1	1.2	0.3–3.9
T-G-T	2%	1%	0.68	1	0.7	0.1–3.2
