# Supplementary material for: Polymorphism rs7278468 is associated with Age-related cataract through decreasing transcriptional activity of the CRYAA promoter
Source: Sci Rep. 2016 Mar 17;6:23206. doi: 10.1038/srep23206 (PMC4794711; doi:10.1038/srep23206)
Supplement: Supplementary Information [file srep23206-s1.doc]

**Polymorphism rs7278468 is associated with Age-related cataract through decreasing transcriptional activity of the *CRYAA* promoter**

Xiaoyin Ma^1, 2^, Xiaodong Jiao^2^, Zhiwei Ma^2^, J. Fielding Hejtmancik^2*^

^1^Laboratory of Developmental Cell Biology and Disease, School of Ophthalmology and Optometry and Eye Hospital, Wenzhou Medical University, 325003, China

^2^Ophthalmic Genetics and Visual Function Branch, National Eye Institute, National Institutes of Health, Bethesda, MD 20892, USA

*Correspondence to: J. Fielding Hejtmancik, MOGS/OGVFB/NEI/NIH, 5635 Fisher’s Lane, Room 1127, Rockville, MD, 20852; Phone: (301) 496-8300; FAX: (301) 435-1598; email: f3h@helix.nih.gov

| **Matrix Family** | **Detailed Family Information** | **Matrix** | **Detailed Matrix Information** | **Tissue** | **Opt.** | **Start position** | **End position** | **Anchor position** | **Strand** | **Core sim.** | **Matrix sim.** | **Mat. sim.**  **-**  **opt.** | **Sequence** |
| --- | --- | --- | --- | --- | --- | --- | --- | --- | --- | --- | --- | --- | --- |
| **V$SP1F** | **GC-Box factors SP1/GC*: CDCA7L, EAPP, KLF10, KLF11, KLF16, KLF5, SP1, SP2, SP3, SP4, SP5, SP6, SP7, SP8** | **V$TIEG.01** | **TGFbeta- inducible early gene (TIEG) /**  **Early growth response gene alpha (EGRalpha)** | **Ubiquitous** | **0.8** | **628** | **644** | **636** | **+** | **1** | **1** | **0.17** | **aacgGGGGtgtgtgctc** |
| **V$BRAC** | **Brachyury gene, mesoderm developmental factor: EOMES, MGA,T, TBR1, TBX1, TBX10, TBX15, TBX18, TBX19, TBX2, TBX20, TBX21, TBX22, TBX23P, TBX3, TBX4, TBX5, TBX6** | **V$TBX21.01** | **T-box transcription factor TBX21,**  **dimeric binding site** | **Blood Cells, Brain Breast, Cardiovascular System, Central Nervous System, Ear, Embryonic Structures, Endocrine System, Heart, Immune System, Leukocytes, Lymphocytes, Muscles, Myocardium, Nervous System, arathyroid Glands**  **Pituitary Gland** | **0.9** | **624** | **652** | **638** | **+** | **1** | **0.89** | **0.02** | **gtctaacgggGGTGtgtgct ctccctcct** |
| **V$KLFS** | **Krueppel like transcription factors^: KLF1, KLF12, KLF13, KLF15, KLF17, KLF2, KLF3, KLF4, KLF6, KLF7, KLF8, KLF9** | **V$BKLF.01** | **Basic krueppel- like factor (KLF3)** | **Blood Cells, Bone Marrow Cells, Embryonic Structures, Erythrocytes, Hematopoietic System** | **1** | **630** | **646** | **638** | **+** | **1** | **0.95** | **0** | **cggGGGTgtgtgctctc** |
| **V$BRAC** | **Brachyury gene, mesoderm developmental factor: EOMES, MGA,T, TBR1, TBX1, TBX10, TBX15, TBX18, TBX19, TBX2, TBX20, TBX21, TBX22, TBX23P, TBX3, TBX4, TBX5, TBX6** | **V$MGA.01** | **MAX gene associated, dimeric binding site** | **Blood Cells, Brain, Breast, Cardiovascular System, Central Nervous System, Ear, Embryonic Structures, Endocrine System, Heart, Immune System, Leukocytes, Lymphocytes, Muscles, Myocardium, Nervous System, Parathyroid Glands, Pituitary Gland** | **0.8** | **631** | **659** | **645** | **-** | **1** | **0.79** | **0.04** | **tcgccagaggagggagagca cACACcccc** |
| **V$BRAC** | **Brachyury gene, mesoderm developmental factor: EOMES, MGA,T, TBR1, TBX1, TBX10, TBX15, TBX18, TBX19, TBX2, TBX20, TBX21, TBX22, TBX23P, TBX3, TBX4, TBX5, TBX6** | **V$MGA.01** | **MAX gene associated, dimeric binding site** | **Blood Cells, Brain, Breast, Cardiovascular System, Central Nervous System, Ear, Embryonic Structures, Endocrine System, Heart, Immune System, Leukocytes, Lymphocytes, Muscles, Myocardium, Nervous System, Parathyroid Glands, Pituitary Gland,** | **0.8** | **633** | **661** | **647** | **-** | **1** | **0.81** | **0.06** | **ggtcgccagaggagggaga gcACACaccc** |
| ***Evidence for GC-Box factors SP1/GC: part of model match of KLFS_SP1F_03,SP1F_EBOX_SP1F_01,SP1F_KLFS_01** | | | | | | | | | | | | | |
| **^Evidence for Krueppel like transcription factors: part of model match of KLFS_SP1F_03**  **Blue: not seen in chick lens fibers, light orange: NE < 1, medium orange: 1 < NE < 10, dark orange: NE >10 Note: SP2 increased from epithelia to fibers, CDCA7L decreased from epithelia to fibers, the remaining factors did not change significantly. NE = 107 * #readsregion / (#readsmapped * lengthregion).** | | | | | | | | | | | | | |

Table S1. Transcription factor binding sites overlapping rs7278468

**Supplemental data: Figures**


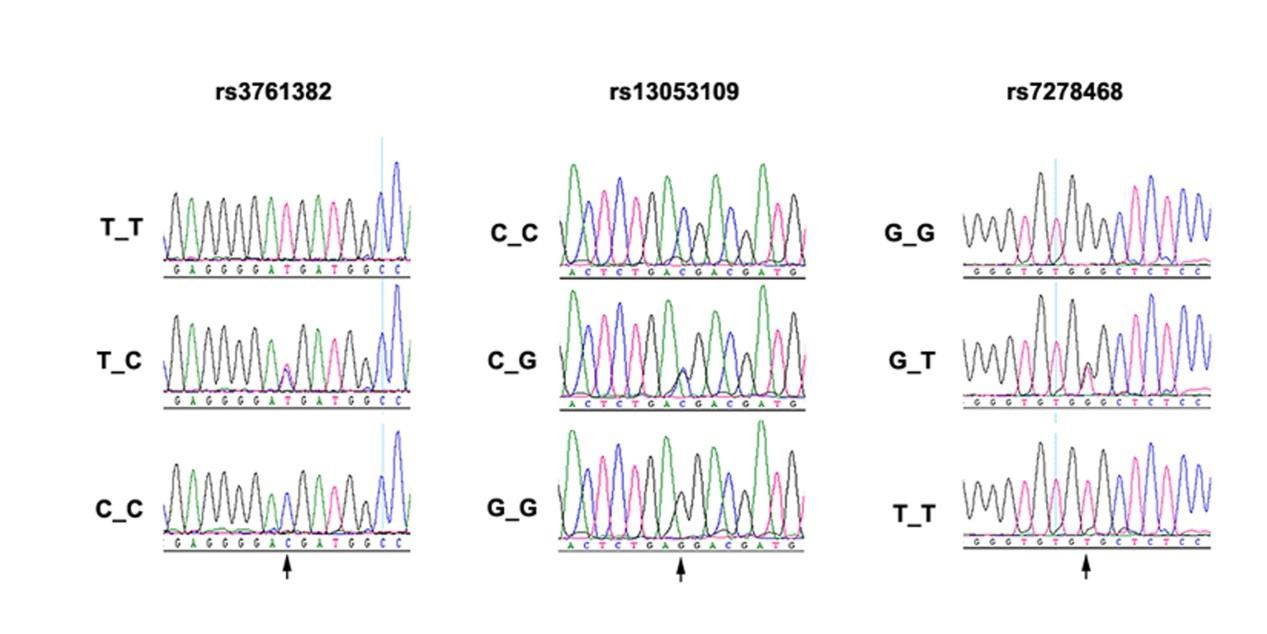


Figure S1. Sequencing results of the three SNP sites in the CRYAA promotor region.


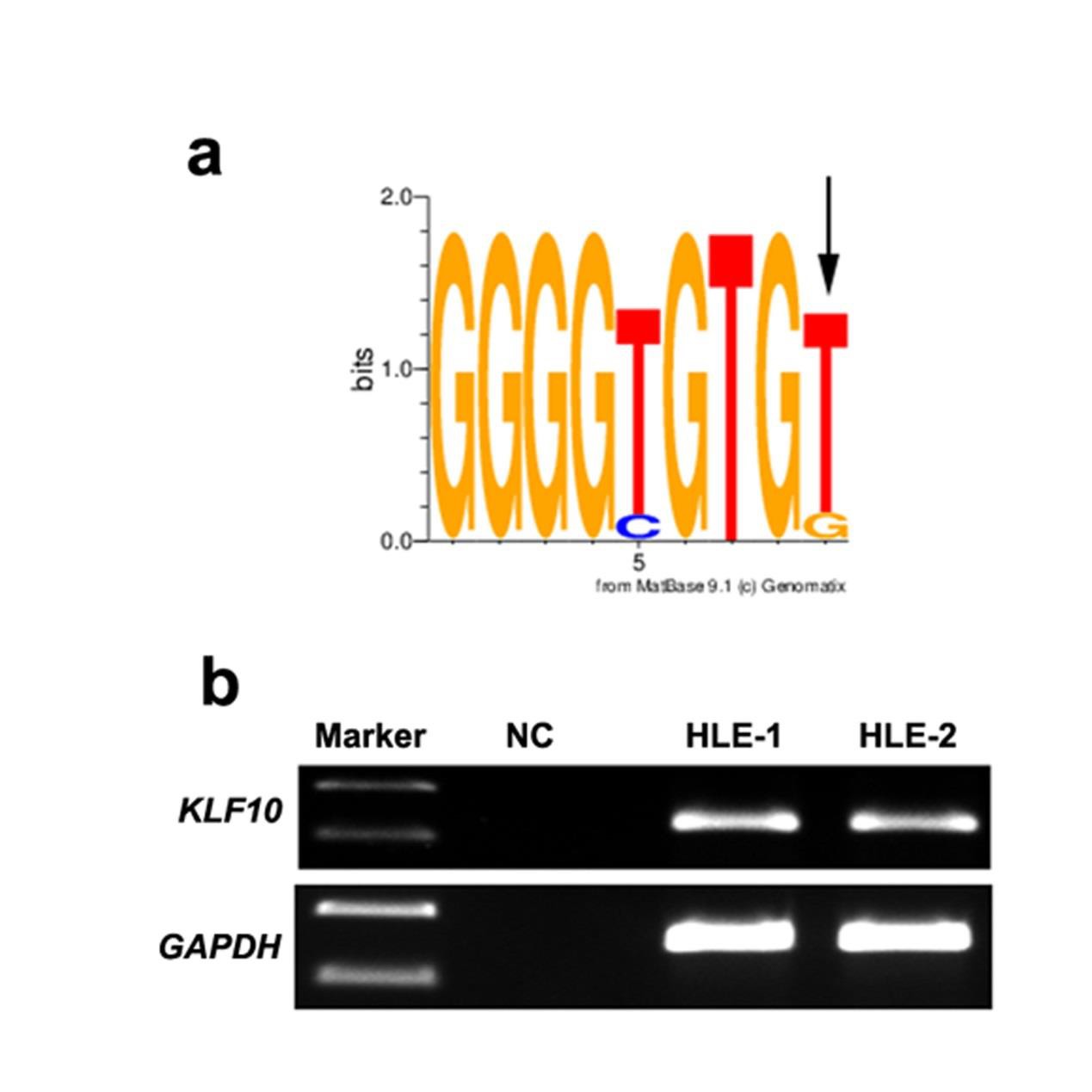


Figure S2, a. Genomatix analysis and prediction of the KLF10 binding motif in *CRYAA* promoter. rs7278468 is marked by the arrow. B. *KLF10* mRNA expression in HLE cells was tested by RT-PCR.


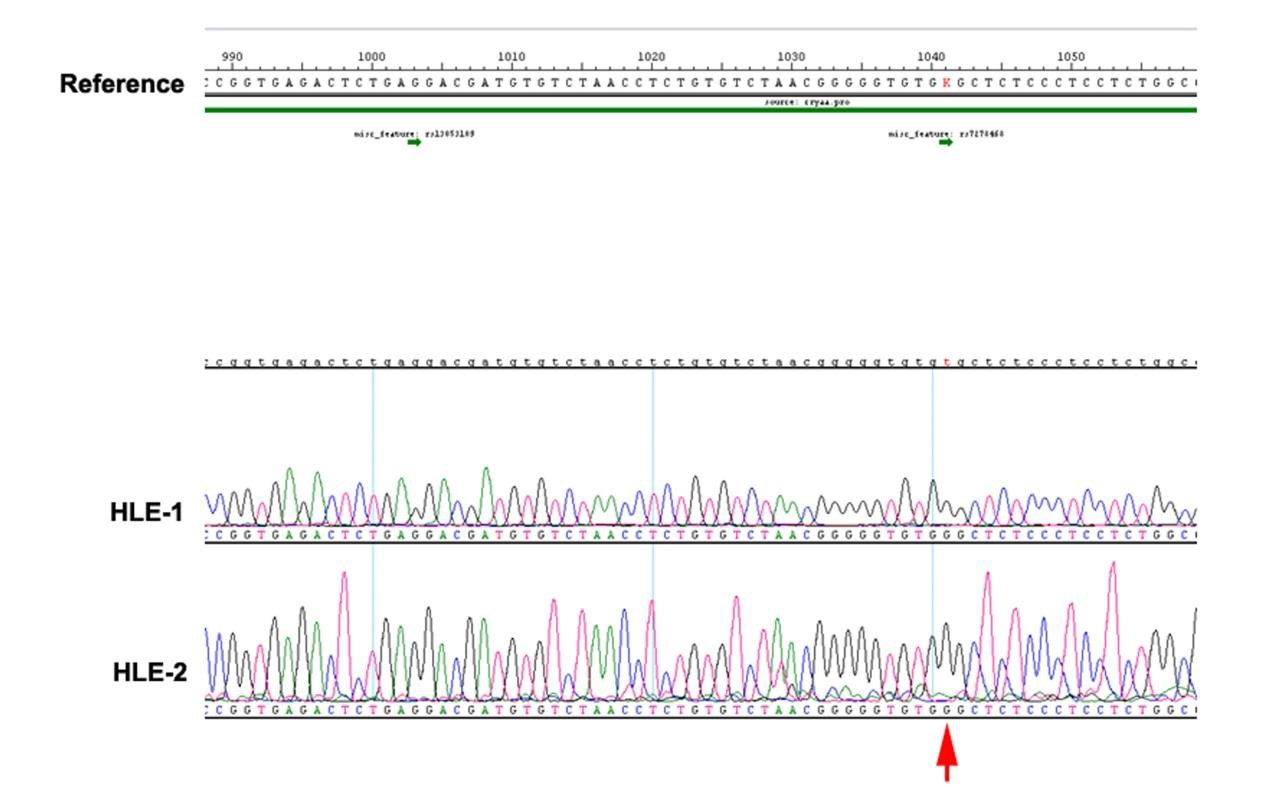


Figure S3. rs7278468 genotype of HLE cells.


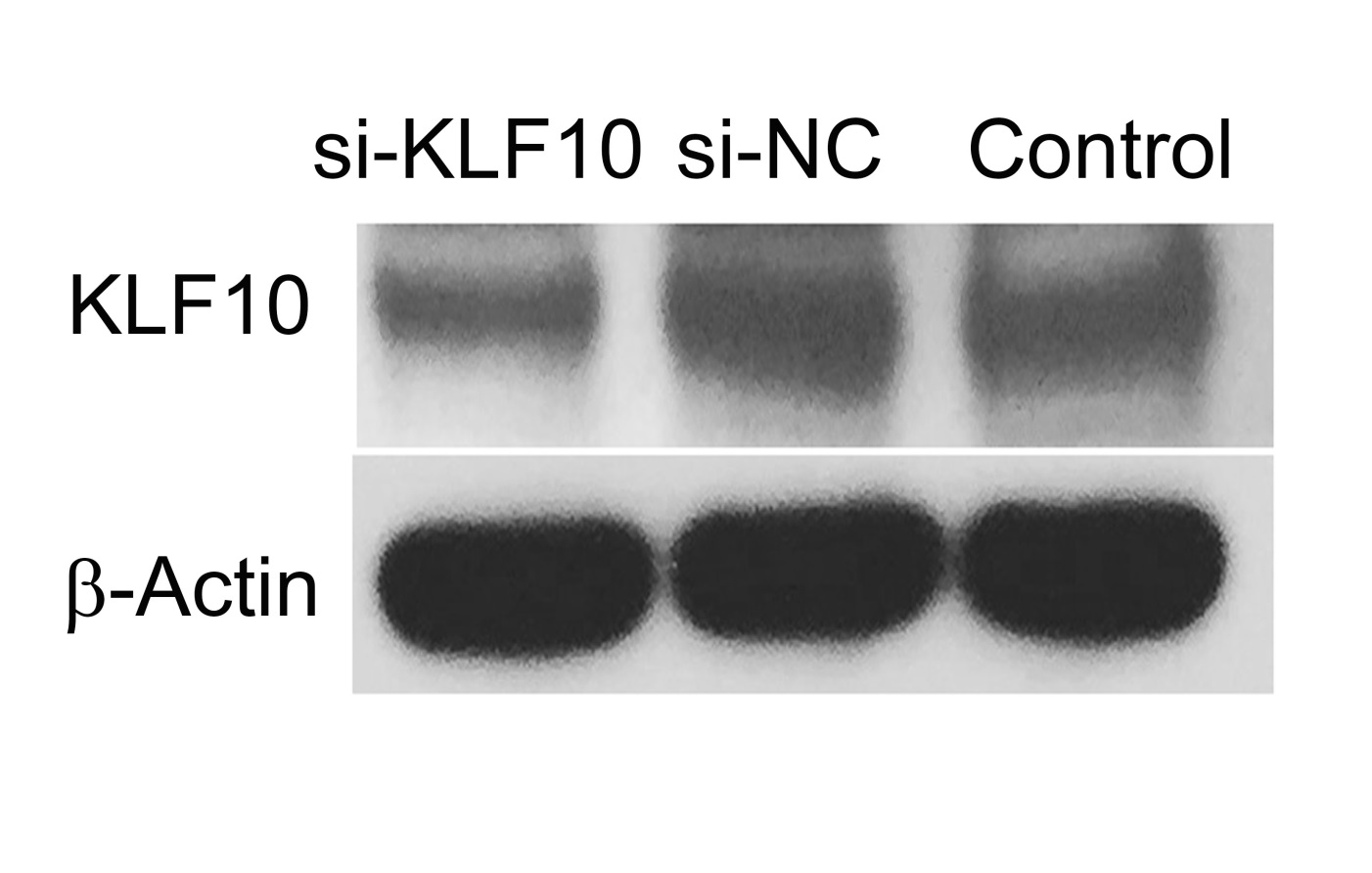


Figure S4. Western blot of KLF10 knockdown by siRNA.


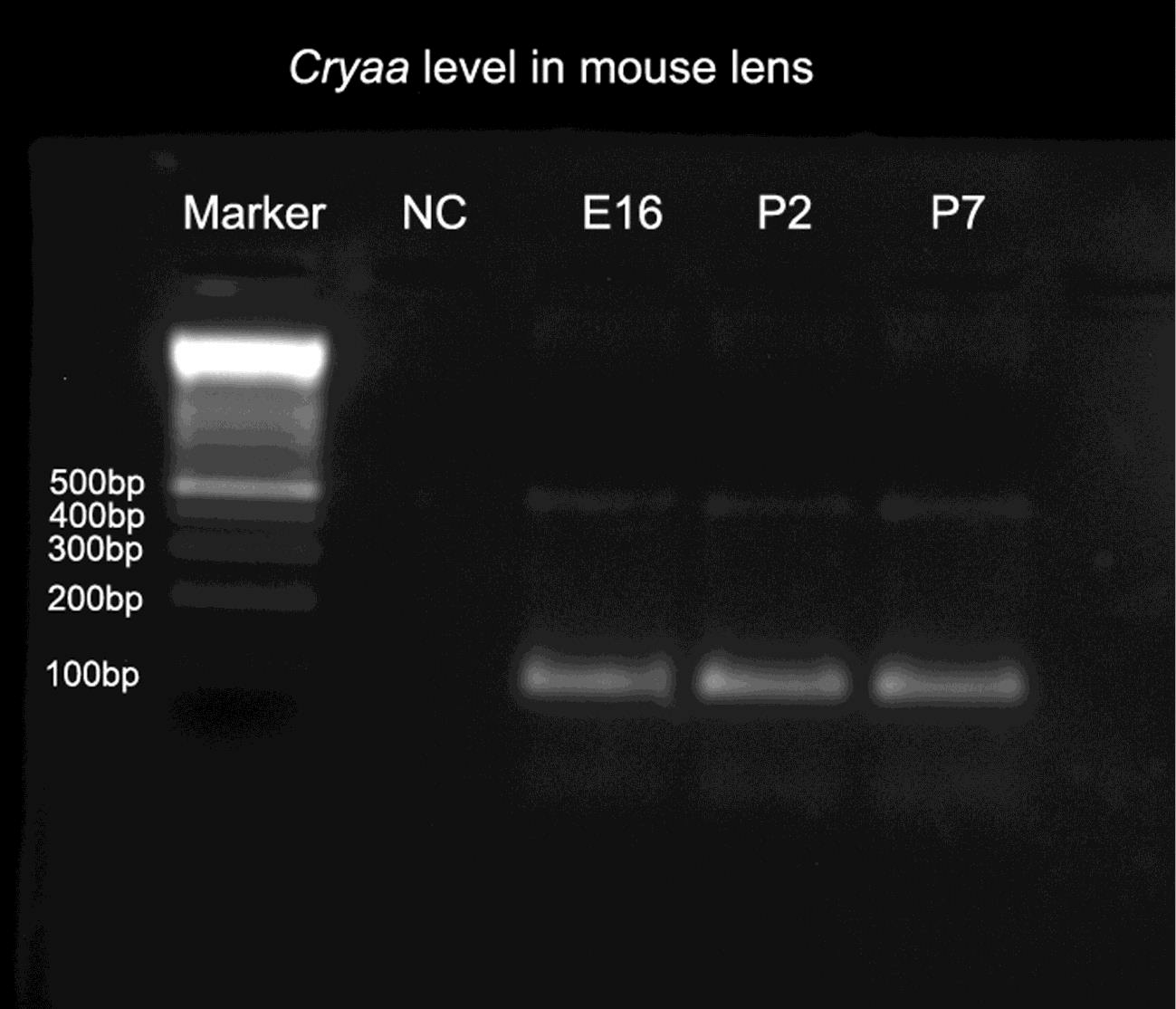


Figure 3a. Cryaa whole gel.


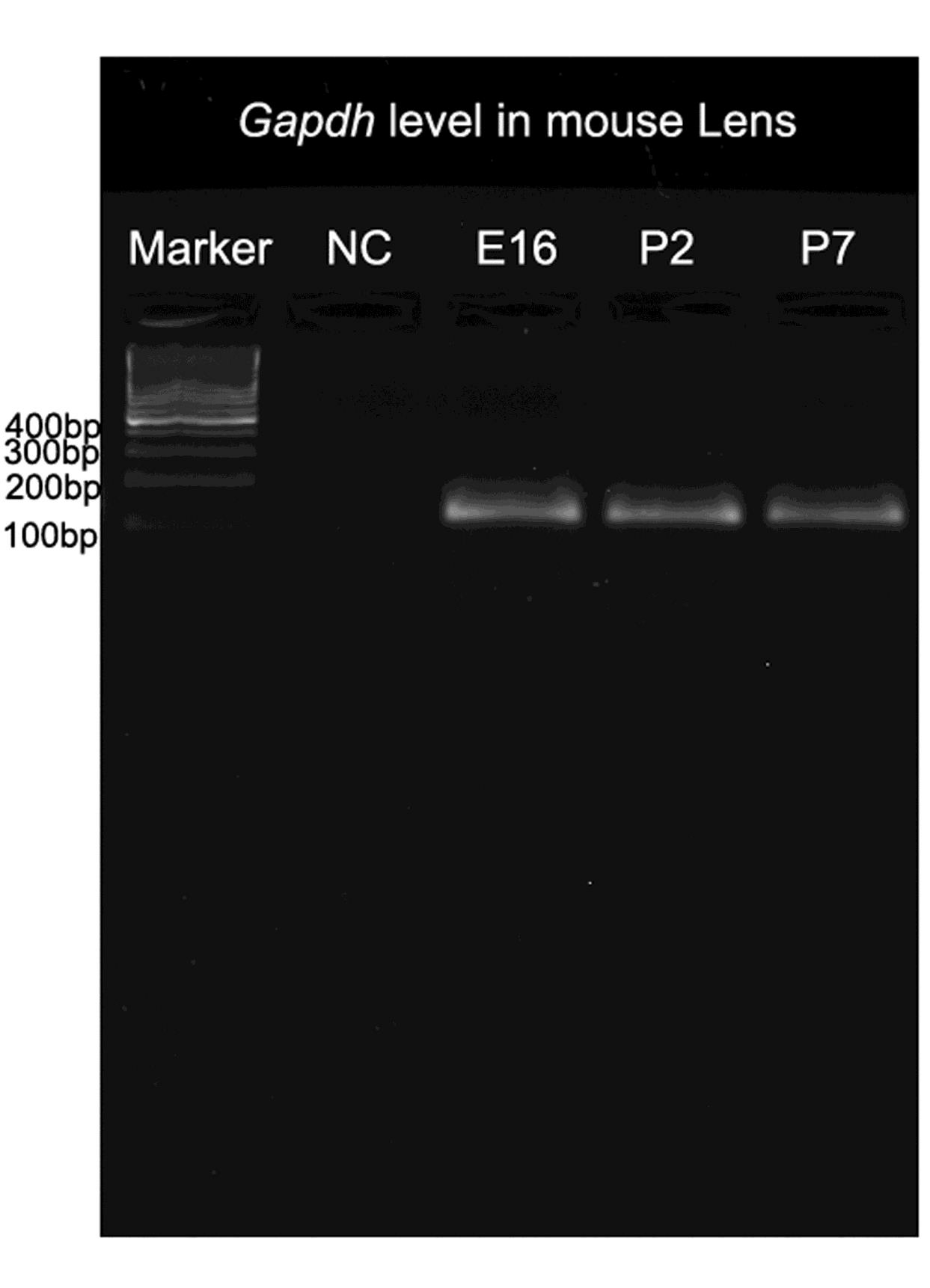


Figure 3a. Gapdh whole gel.


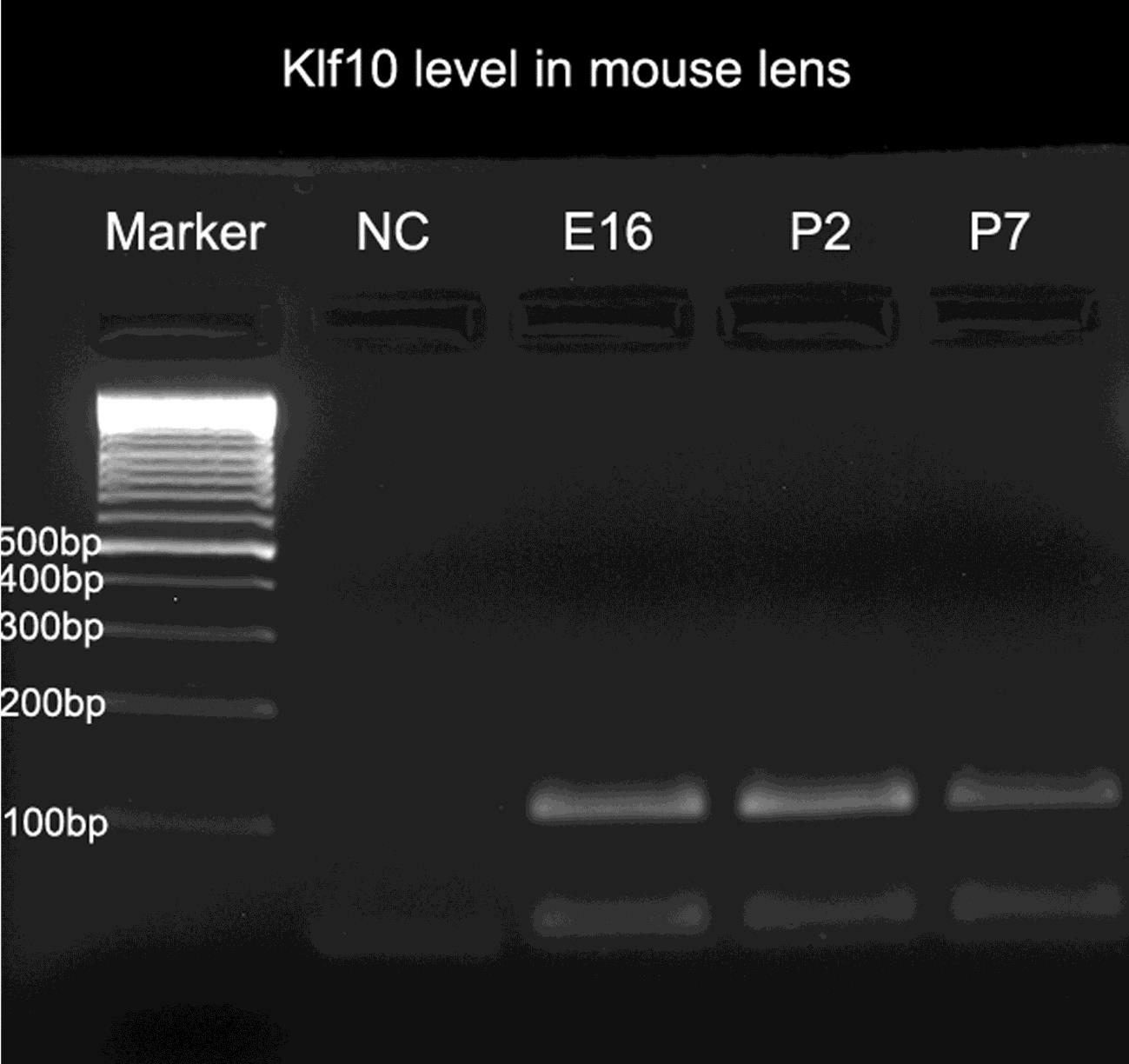


Figure 3a. Klf10 whole gel.


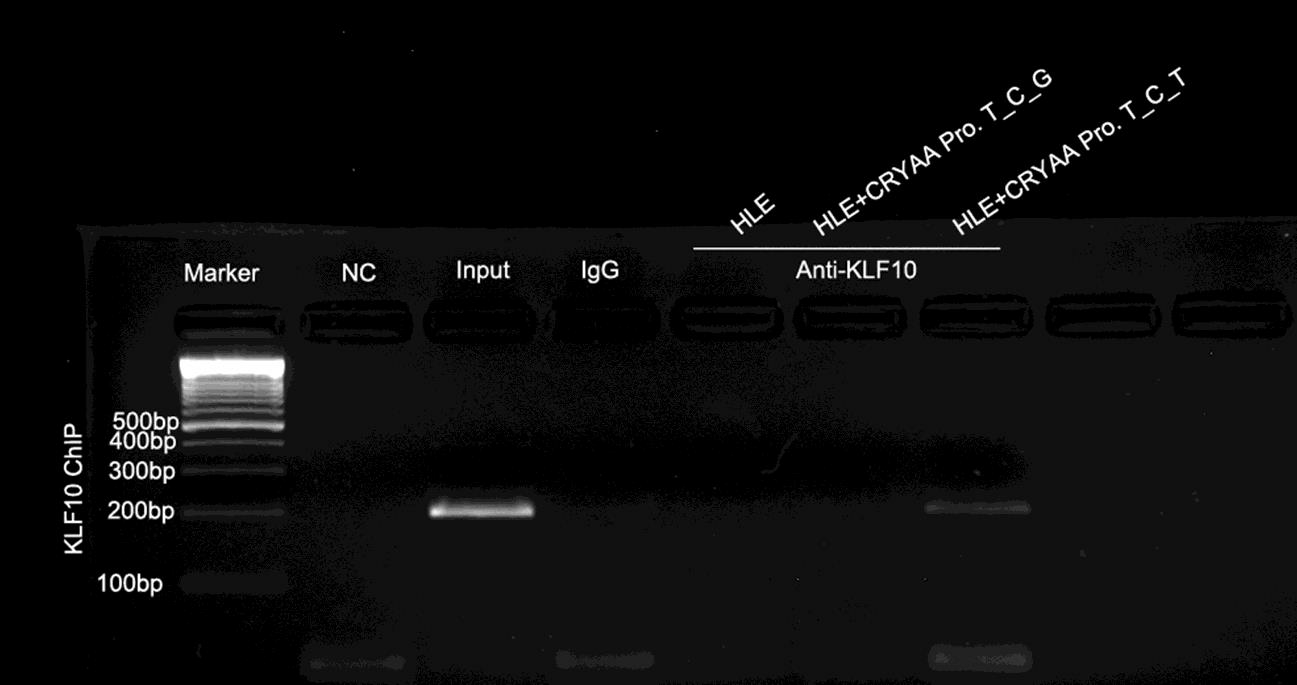


Figure 3c. KLF10 ChIP whole gel.


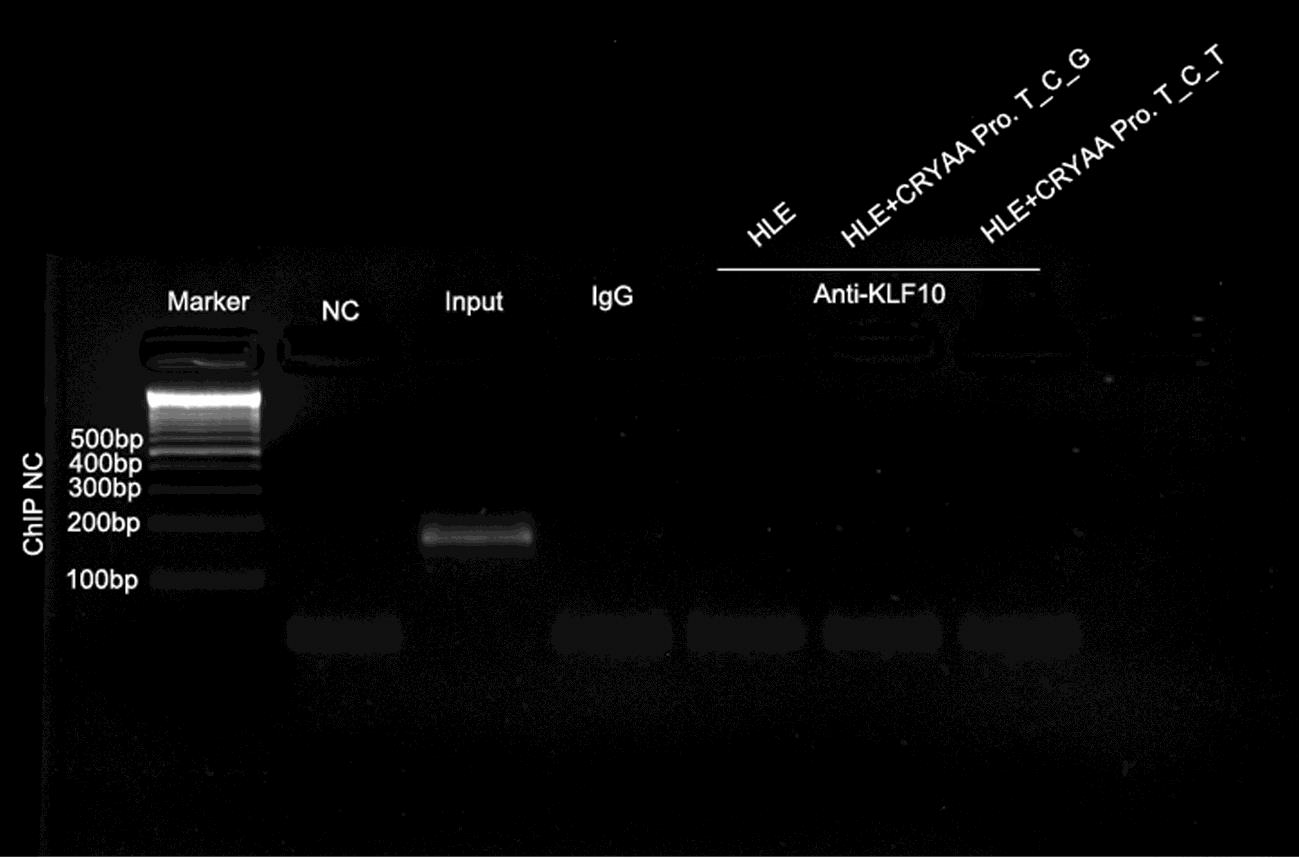


Figure 3c. NC ChIP whole gel.


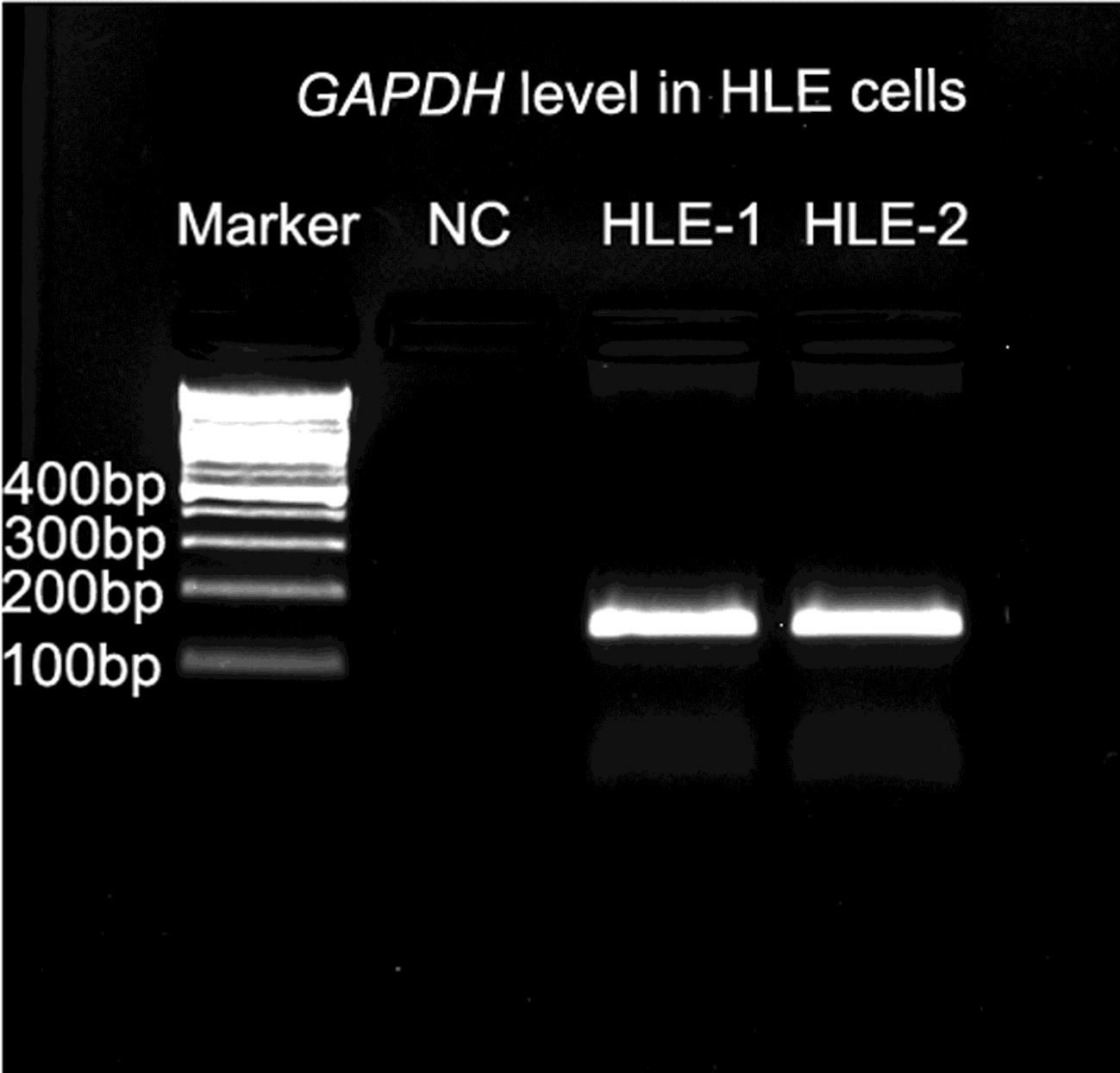


Figure S2b. GAPDH in HLE whole gel.


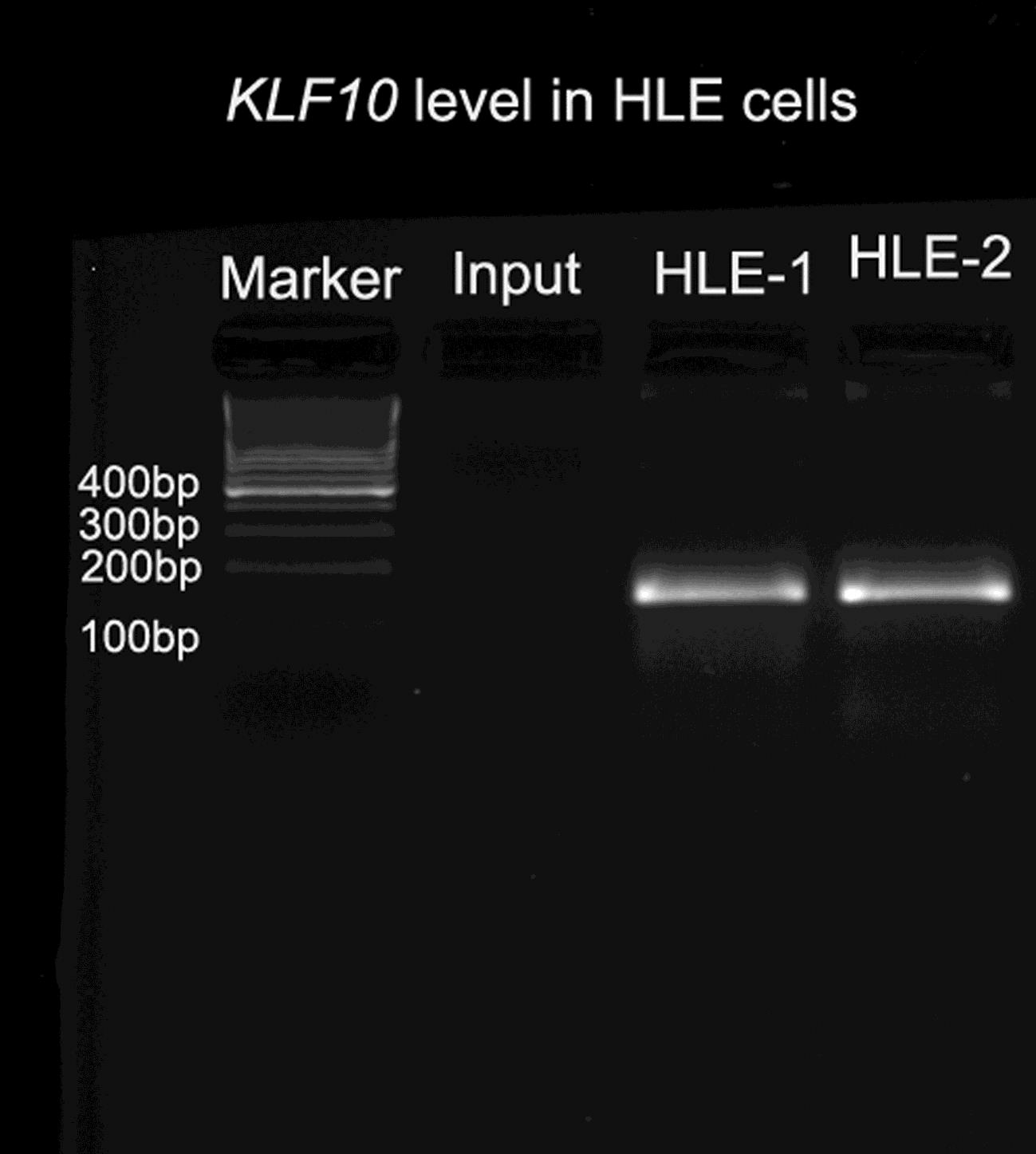


Figure S2b. KLF10 in HLE whole gel.


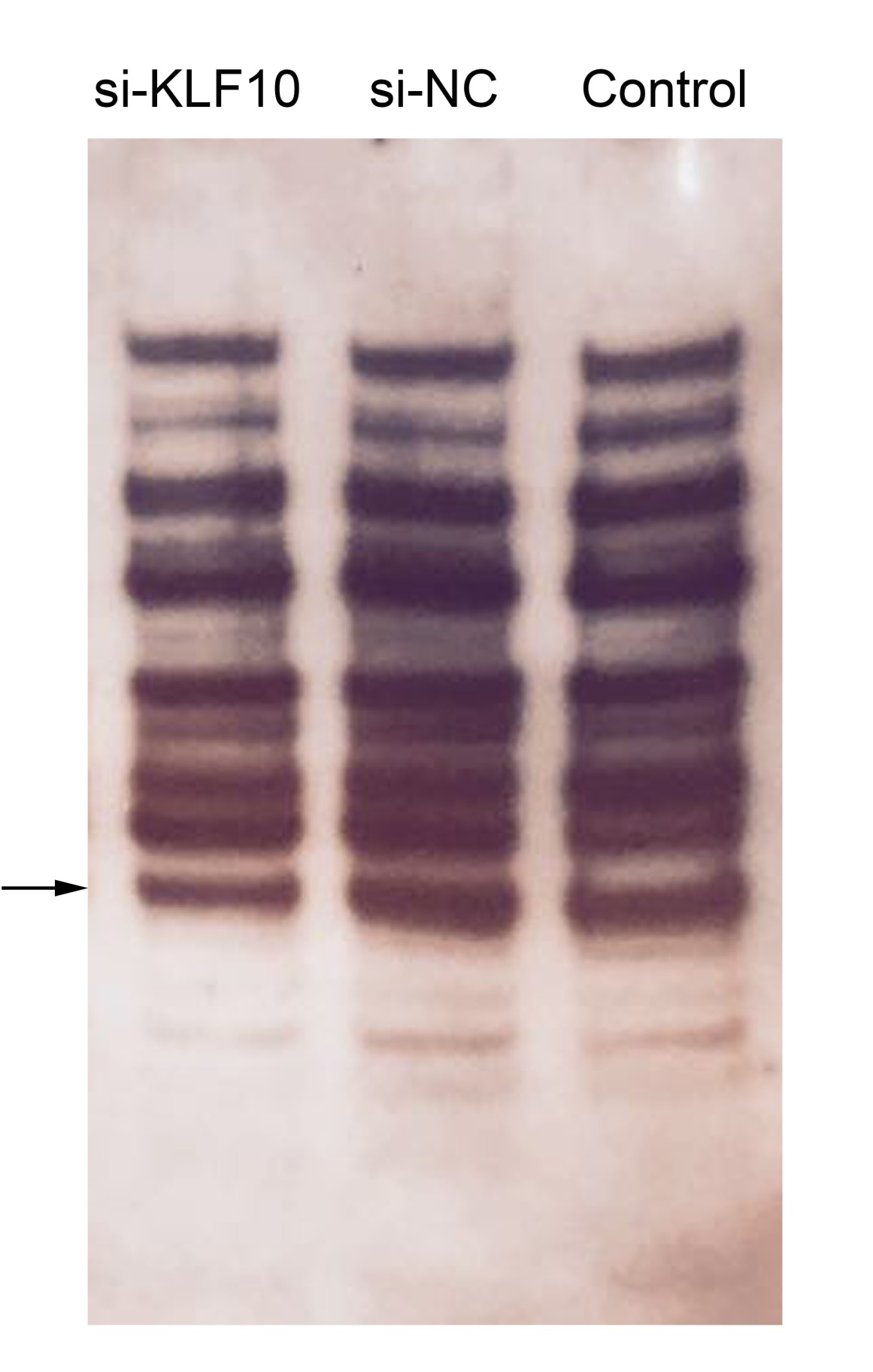


Figure S4. KLF10 si-RNA knockdown in HLE Western whole gel. Arrow indicates molecular mass of KLF10.


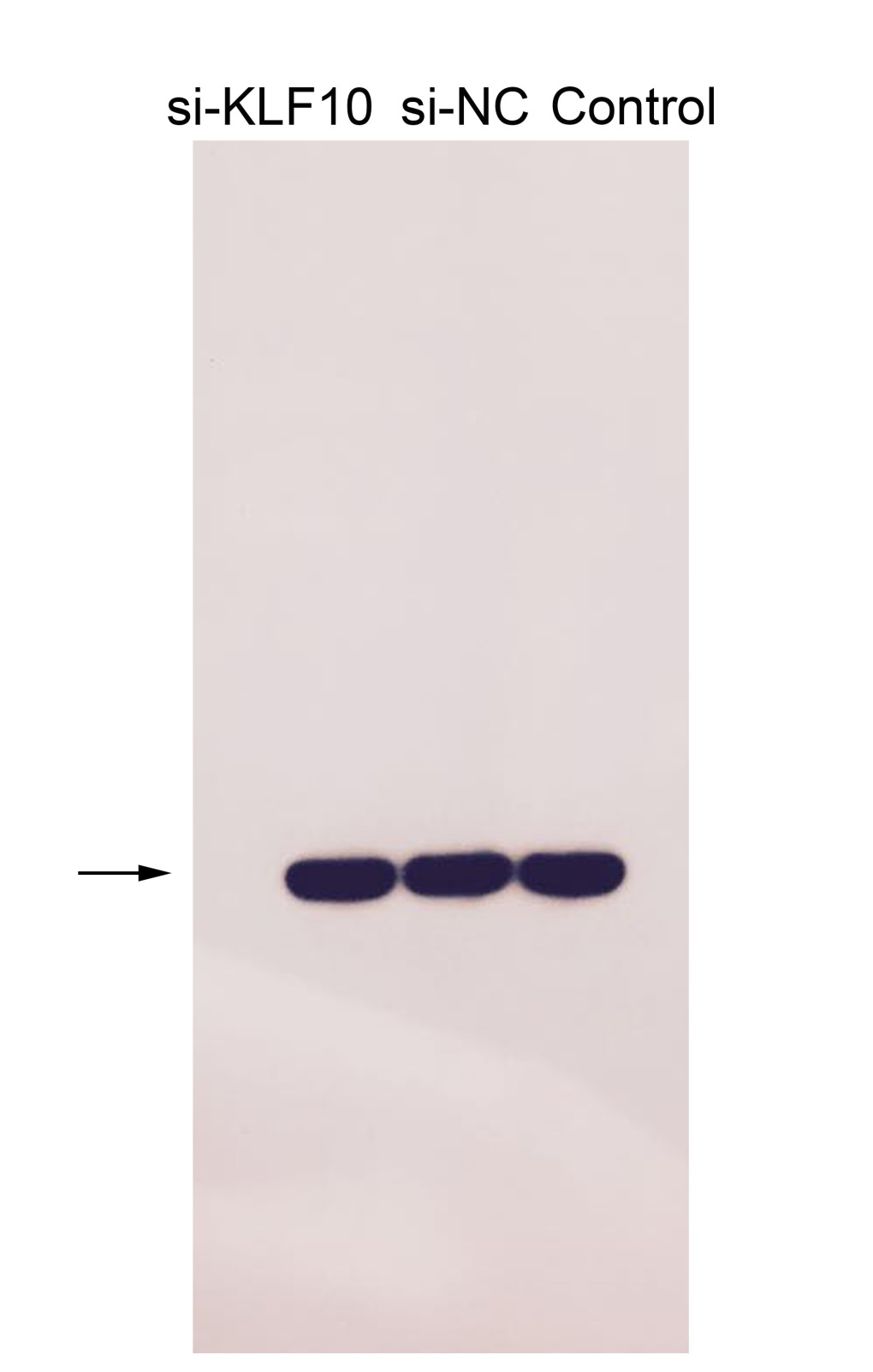


Figure S4. Β-Actin si-RNA knockdown in HLE Western whole gel. Arrow indicates molecular mass of β-actin.
